# Supplementary material for: Metabolic Pathway Engineering Improves Dendrobine Production in Dendrobium catenatum
Source: Int J Mol Sci. 2023 Dec 28;25(1):397. doi: 10.3390/ijms25010397 (PMC10778673; doi:10.3390/ijms25010397)
Supplement: Supplementary file 1 [file ijms-25-00397-s001.zip › ijms-2787291-supplementary.pdf]

Metabolic pathway engineering improves dendrobine production in *Dendrobium catenatum*

Meili Zhao 1,2,†, Yanchang Zhao 3,†, Zhenyu Yang 1,4, Feng Ming 4, Jian Li 1,2, Demin Kong 1,2, Yu Wang 1,2, Peng Chen 1,2, Meina Wang 1,2,* and Zhicai Wang 1,2,*

1 Shenzhen Key Laboratory for Orchid Conservation and Utilization, The National Orchid Conservation Center of China and the Orchid Conservation & Research Center of Shenzhen, Shenzhen 518114, China; 17720798949@163.com (M.Z.); yangzhenyu.eco@foxmail.com (Z.Y.); lee799908@hotmail.com (J.L.); damykong@126.com (D.K.); 15361439572@163.com (Y.W.); chenpeng1003@163.com (P.C.)

2 Key Laboratory of National Forestry and Grassland Administration for Orchid Conservation and Utilization, The National Orchid Conservation Center of China and the Orchid Conservation & Research Center of Shenzhen, Shenzhen 518114, China

3 State Key Laboratory for Conservation and Utilization of Subtropical Agro-Bioresources, South China Agricultural University, Guangzhou 510642, China; 13965002218@163.com

4 Shanghai Key Laboratory of Plant Molecular Sciences, College of Life Sciences, Shanghai Normal University, Shanghai 200234, China; fming@fudan.edu.cn

* Correspondence: wangmn@sztcg.com (M.W.); wangzc@cnocc.cn (Z.W.);

Tel.: +86-0755-25712047 (M.W.); +86-0755-25723979 (Z.W.)

† The authors contributed equally to this work.

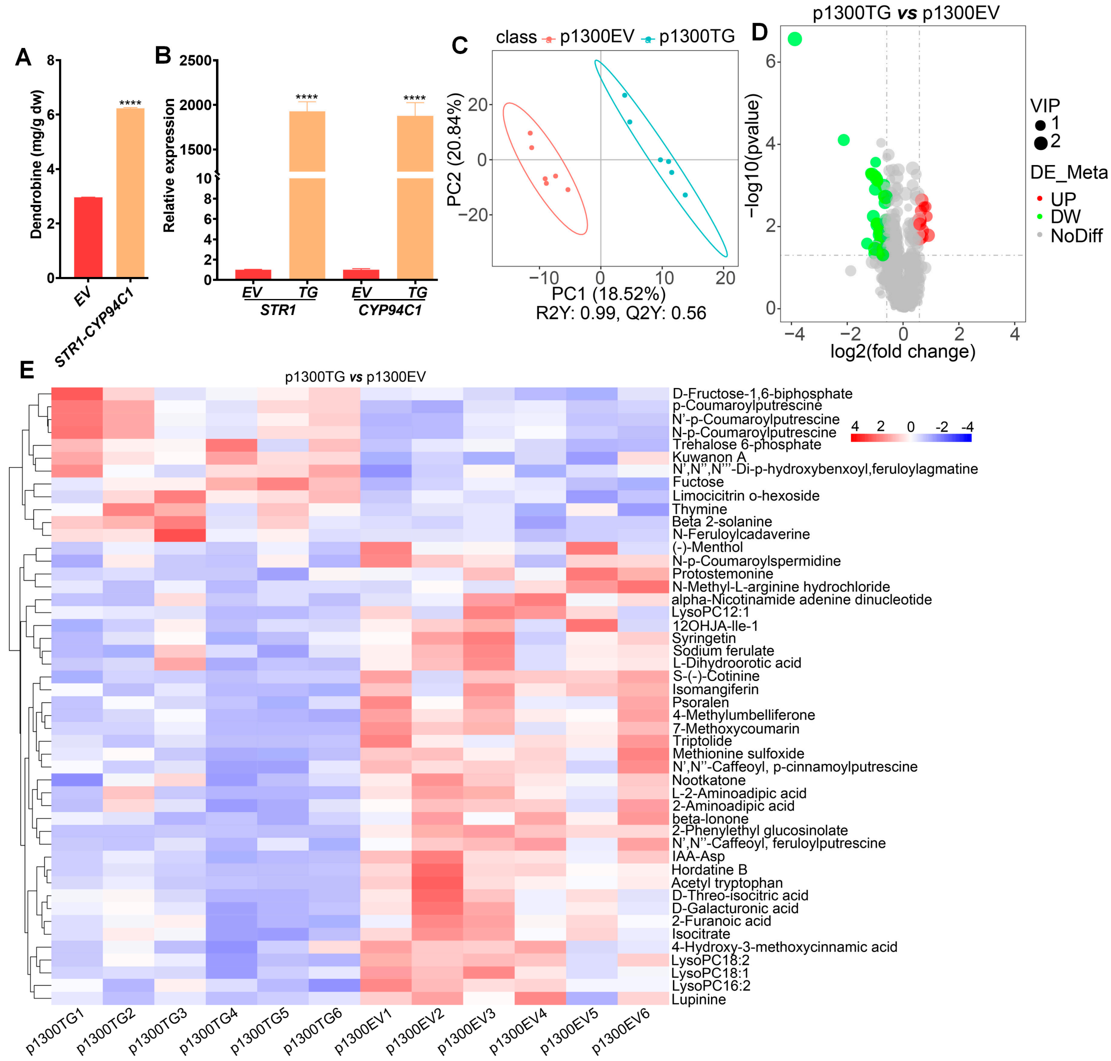

Figure S1. *STR1-CYP94C1* (*TG*) enhanced dendrobine production in *Dendrobium catenatum* leaves but not in *Nicotiana benthamiana* leaves. (A) Dendrobine accumulation in *D. catenatum* leaves infiltrated with *Agrobacterium* carrying pCambia1300-*STR1-CYP94C1*. (B) qRT-PCR expression analysis of *STR1* and *CYP94C1* in *N. benthamiana* leaves infiltrated with *Agrobacterium* carrying pCambia1300-*STR1-CYP94C1*. (C) The partial least squares-discriminant analysis (PLS-DA) score plots based on the GC-MS data of *TG*-expressing *N. benthamiana* leaves in comparison with empty vector controls (n = 6). (D) Variable importance for projection (VIP) plot showing the differential metabolites in *TG*-expressing *N. benthamiana* leaves compared to empty vector controls. (E) A heatmap hierarchical clustering of the differentially accumulated metabolites (DAMs) in *TG*-multigene expressing leaves of *N. benthamiana*. The log2 of the metabolite quantification was used. The columns indicate the *TG*-multigene and the empty vector control expressing samples, each with six replicates, while the rows correspond to the different metabolites. Statistical significance in (A) and (B) was demonstrated as **** $P \leq 0.0001$.

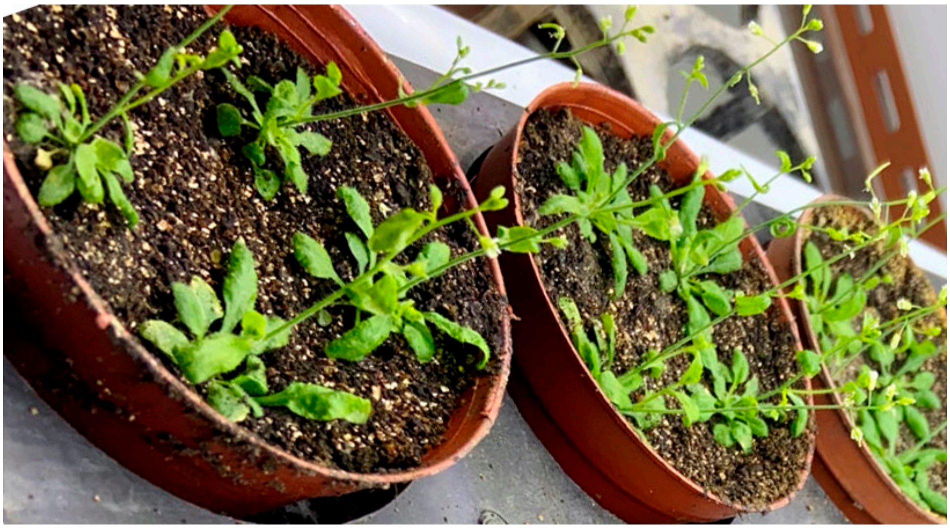

EV *SG*

Figure S2. Representative images of earth-growing *Arabidopsis* transgenic plants. Image showed plants growing in earth pots for 1 month. *EV*: empty vector. *SG*: seven genes.

Table S1. Cellular localization prediction of proteins

Proteins	Location	LocDB	PotLocDB	Neural Nets	Pentamers	Integral
CMK	Chloroplast	0.0	5.0	0.00	3.24	8.84
DXR	Chloroplast	10.0	3.0	0.00	5.24	9.29
MCT	Chloroplast	0.0	5.0	0.00	1.91	9.19
STR1	Plasma membrane	10.0	0.0	0.96	0.83	9.49
CYP94C1	Plasma membrane	0.0	2.9	0.91	0.48	5.93
BCAT2	Cytoplasmic	3.2	0.0	0.0	0.32	2.61
METTL23	Extracellular	0.0	0.0	0.95	2.14	2.46
CMEAO	Extracellular	0.0	0.0	0.92	1.76	2.43
MYB61	Nuclear	5.0	3.0	0.00	0.13	8.28

Table S2. Primers used in this study

Vector construction primers		
DXR	Forward	5'-ATGGCGTTGAAGCTGCCAT-3'
	Reverse	5'-CTAAGCAGGAACTGGACTCAATCC-3'
MCT	Forward	5'-ATGATGGCACTCCCATATCAGC-3'
	Reverse	5'-TCATGTTTCCATGTTTATTATCCTCTCT-3'
CMK	Forward	5'-ATGGCTTCTGTGTCCAACCAC-3'
	Reverse	5'-TTATTCTCCTGTTTGTGACATCTCTTC-3'
MET	Forward	5'-ATGAGTACTCGATCTATAGAAATTGCTG-3'
	Reverse	5'-CTAATCGCGATAGATAATGAACACAG-3'
BCAT2	Forward	5'-ATGTTGACAAGAAGAATTCCTCAAA-3'
	Reverse	5'-TTAGTCAATCTCAACCGTCCATC-3'
MYB61	Forward	5'-ATGGGAAAGCAATCCTACTTACAC-3'
	Reverse	5'-CTAAGACTGCAGCTGCGGC-3'
CMEAO	Forward	5'-ATGGCCGCAGCTGAGGA-3'
	Reverse	5'-TCACAACTTACTGAGCAAAACATTCT-3'
STR1	Forward	5'-ATGGCACTCGCTGGGGC-3'
	Reverse	5'-TTACTCTAATGTAAATACGGCTAT-3'
CYP94C1	Forward	5'-ATGGAACAAGTCGTCTCCTTTCTC-3'
	Reverse	5'-TCATCTCCTCTTGCTCACTCGA-3'
qRT-PCR primers		
qAtActin	Forward	5'-CACTTGCACCAAGCAGCATGAAGA-3'
	Reverse	5'-AATGGAACCACCGATCCAGACACT-3'
qSTR1	Forward	5'-TCAACTTTCAGAGGAGGCACTTC-3'
	Reverse	5'-CTTGTCACCTTTCAGCCAGTATCT-3
qCYP94C1	Forward	5'-GCCTTCATCTCCACCTCCTTC-3'
	Reverse	5'-AGGTGAGTGTACCAATCGCAGA-3'
qDXR	Forward	5'-TCATAGAGGTCGCATGGCATC-3'
	Reverse	5'-TTGTGTTTATGTGCAAGGGGAA-3'
qMETTL23	Forward	5'-TGGTGCTGATGTCGGATTTG-3'
	Reverse	5'-CTTCCGGCGATCTCATTAGC-3'
qDcActin	Forward	5'-GAAGCCCAGTCCAAAAGAGGTATCC-3'
	Reverse	5'-ACATGGCAGGCACATTGAAAGTCTC-3'
qNbActin7	Forward	5'-CGTCTGTGATAATGGGACTGGA-3'
	Reverse	5'-CATCCCAGTTGCTAACAATACCAT-3'
qBCAT2	Forward	5'-CGAAAGCTATCGTGCAACTCC-3'
	Reverse	5'-CTTTCGGATAAATATGTTGCAGGA-3'
qCMK	Forward	5'-CTAGAGCCCCCTGCATTTGA-3'

	Reverse	5'-TAAACAAATTGAGGTGGGTCTGG-3'
qMCT	Forward	5'-TGGTGTTCCTGTAAAGGCTACTATT-3'
	Reverse	5'-TGTTCCAAGTGTTCCACGATAGA-3'
qMYB61	Forward	5'-CCTGAGGAGGATGAGAAGTTGC-3'
	Reverse	5'-AACTGTGCTGCAATCTGAGACC-3'
qCMEAO	Forward	5'-GAATAGGTCACTTGAGGAAACGG-3'
	Reverse	5'-TCAATGTCACTAGGCGGCAC-3'
